# Structure of the trypanosome paraflagellar rod and insights into non-planar motility of eukaryotic cells

**DOI:** 10.1038/s41421-021-00281-2

**Published:** 2021-07-13

**Authors:** Jiayan Zhang, Hui Wang, Simon Imhof, Xueting Zhou, Shiqing Liao, Ivo Atanasov, Wong H. Hui, Kent L. Hill, Z. Hong Zhou

**Affiliations:** 1 Department of Microbiology, Immunology and Molecular Genetics, University of California, Los Angeles (UCLA), Los Angeles, CA USA; 2 Molecular Biology Institute, UCLA, Los Angeles, CA USA; 3 California NanoSystems Institute, UCLA, Los Angeles, CA USA; 4 Department of Bioengineering, UCLA, Los Angeles, CA USA

**Keywords:** Cell migration, Cryoelectron tomography

## Abstract

Eukaryotic flagella (synonymous with cilia) rely on a microtubule-based axoneme, together with accessory filaments to carryout motility and signaling functions. While axoneme structures are well characterized, 3D ultrastructure of accessory filaments and their axoneme interface are mostly unknown, presenting a critical gap in understanding structural foundations of eukaryotic flagella. In the flagellum of the protozoan parasite *Trypanosoma brucei* (*T. brucei*), the axoneme is accompanied by a paraflagellar rod (PFR) that supports non-planar motility and signaling necessary for disease transmission and pathogenesis. Here, we employed cryogenic electron tomography (cryoET) with sub-tomographic averaging, to obtain structures of the PFR, PFR-axoneme connectors (PACs), and the axonemal central pair complex (CPC). The structures resolve how the 8 nm repeat of the axonemal tubulin dimer interfaces with the 54 nm repeat of the PFR, which consist of proximal, intermediate, and distal zones. In the distal zone, stacked “density scissors” connect with one another to form a “scissors stack network (SSN)” plane oriented 45° to the axoneme axis; and ~370 parallel SSN planes are connected by helix-rich wires into a paracrystalline array with ~90% empty space. Connections from these wires to the intermediate zone, then to overlapping layers of the proximal zone and to the PACs, and ultimately to the CPC, point to a contiguous pathway for signal transmission. Together, our findings provide insights into flagellum-driven, non-planar helical motility of *T. brucei* and have broad implications ranging from cell motility and tensegrity in biology, to engineering principles in bionics.

## Introduction

Eukaryotic cells depend on flagella (synonymous with cilia^1^) to move through and respond to their external environment. In humans, flagellum motility and signaling are essential for normal development, physiology, and reproduction^2–4^. In protists and fungi, flagella enable navigation through diverse environments^1,5–8^, direct movement and interaction of gametes for reproduction^9,10^, and contribute to transmission and pathogenesis of microbial pathogens^11–14^. The structural foundation of the flagellum is the axoneme, a microtubule-based molecular machine that drives motility and provides a platform for assembly of signaling machinery^15^. In addition to the axoneme, flagella of many organisms contain accessory structures, such as outer dense fibers and fibrous sheath of human sperm^16^, mastigonemes of algae^17^, and the paraflagellar rod (PFR) of euglenoids and kinetoplastids^18,19^. Biochemical and genetic analyses have demonstrated that these extra-axonemal structures contribute to flagellum motility and signaling functions, but the structural foundation for how they achieve this is unclear^17,20–22^. Recent studies have resolved structures of the axoneme and axoneme subcomplexes in great detail, providing important insights into mechanisms and structural foundations of flagellum function^23–28^. However, much less is known about 3D ultrastructures of extra-axonemal filaments, and this presents a gap in understanding structural foundations of flagellum function in eukaryotes.

**Fig. 1:**
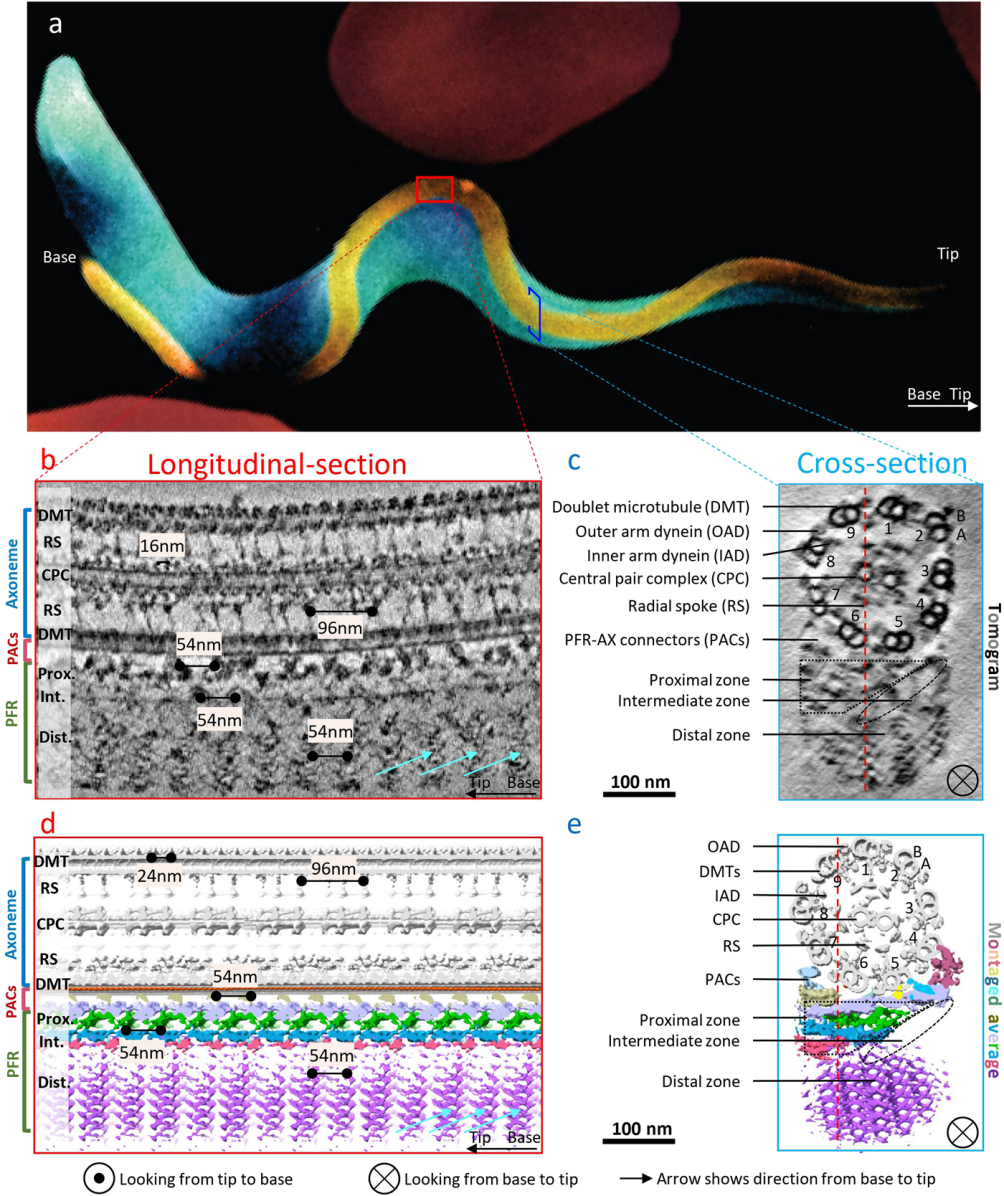
CryoET of *T. brucei* flagellum in its bloodstream form. **a** A scanning EM image of a trypanosome cell (blue) with flagellum (gold) adjacent to red blood cells (red). Artistic rendering based on ref. ^102^. **b**, **c** Longitudinal (**b**) and cross (**c**) sections density slices (10 nm thickness) of a tomogram of the *T. brucei* axoneme and PFR. Arrows in **b** point to apparent “comb teeth” features in the PFR distal zone. **d**, **e** Shaded surface views of longitudinal (**d**) and cross (**e**) sections of a 1248 nm portion of the *T. brucei* axoneme and PFR, obtained by montaging sub-tomographic averages of the axoneme (96 nm repeat), PACs, PFR proximal and distal zones (54 nm repeat). Symbols indicating orientation are defined at the bottom and are used throughout the figures.

Among the most enigmatic of extra-axonemal structures is the PFR of euglenoids and kinetoplastids, a taxonomic group that includes several human and animal pathogens, such as *T. brucei* (Fig. 1a) and related kinetoplastid parasites, as well as free-living *Euglena* and related species^18,19^. *T. brucei* causes fatal sleeping sickness in humans and related diseases in livestock throughout sub-Saharan Africa, while other kinetoplastid parasites cause Chagas disease in the Americas and Leishmaniasis in tropical and subtropical regions globally^29^. The *T. brucei* PFR is a massive paracrystalline filament that runs parallel to the axoneme along most of its length and is connected to axoneme doublet microtubules (DMTs) 4-7^19,30,31^. The exact function of the PFR is not known, but it is required for cell motility, and studies of mutants lacking major portions of the PFR suggest it provides elastic resistance to axoneme bending^20,21,32^. Such internal resistance would be required for efficient movement in viscous environments where an organism must push against high external resistance, e.g., blood and other tissues encountered by *T. brucei*^5,33^. *T. brucei* motility is characterized by a vigorous, non-planar helical motion that must accommodate frequent flagellum beat reversals and collisions with external structures^5,34–36^. Therefore, the PFR must have flexibility while maintaining structural integrity. The PFR also provides a platform for cAMP and Ca^++^ signaling systems that control motility and host–pathogen interactions^13,37–42^, and for metabolic activities that may participate in energy transfer within the flagellum^42,43^. Trypanosome motility and PFR-dependent cAMP signaling are required for transmission and pathogenesis of these deadly pathogens^11–13,39^. Therefore, the PFR presents both a model for understanding functions of extra-axonemal structures of eukaryotic flagella, and an attractive drug target in a group of organisms that pose a substantial global public health burden.

Proteomic and biochemical analyses have provided information on PFR composition^40,44–46^ and conventional electron microscopy and early tomography studies have provided a low resolution model for PFR structure^30,31,47–50^. However, high-resolution 3D structures of the PFR and the PFR-axoneme interface are not available. Consequently, how the PFR and axoneme combine to direct and regulate the hallmark non-planar helical movement of *T. brucei*^5,35,36^ remains a mystery.

Here, we have employed a combination of cryoET with Volta phase plate (VPP), energy filtering, and direct electron-counting imaging, together with sub-tomographic averaging^51^, to determine the structure of the entire *T. brucei* axoneme with PFR. The sub-tomographic averaged structure of the PFR distal zone reveals a largely hollow architecture comprised of planar networks of stacked “scissors densities” placed each 54 nm along and oriented at 45° to the axoneme axis and connected by thin “wire densities”. Such an architecture suggests tensegrity^52^, rather than space-filling observed in other cellular structures^53^, as the means to achieve competing needs of integrity and flexibility. Structural features of wire densities are reminiscent of bundled helices, consistent with secondary structure predictions of major PFR proteins. Based on the sub-tomographic averaged structure of the PFR-axoneme interface and interconnections within the PFR, our work also provides details on interconnections within and between the PFR and axoneme that may provide a means for signaling within these complexes suggested previously^24,27,45,54^. Together, our results provide insights into flagellar motility of *T. brucei* and have broad implications regarding functions of extra-axonemal filaments that are a common, yet enigmatic feature of eukaryotic flagella.

## Results

### Resolving *T. brucei* flagellum components with different periodicities

Presence of the PFR in *T. brucei* flagellum poses two major challenges for structural studies: possible structure distortion due to increased sample thickness and restricted flagellum orientation on the cryoEM grid due to deviation from circularity. To cope with these challenges, we developed a procedure to evaluate the width of the axoneme prior to recording^26^ and relied on sub-tomographic averaging with wedge-mask differences^55^. We also used a machine learning-assisted method to compensate for the missing wedge problem (Materials and Methods). Tilt-series of *T. brucei* detergent-extracted flagellum samples from bloodstream form parasites were recorded in a Titan Krios electron microscope equipped with a VPP, an energy filter and a direct electron detector in electron-counting mode. Tomograms (Fig. 1b, c) were assembled as described in methods. The structure was well-preserved as indicated by presence of all major flagellum components, including the “9 + 2” axoneme, extra-axonemal PFR, and PFR-axoneme connectors (PACs) (Fig. 1b, c; Supplementary Fig. S1 and Supplementary Movie S1). Asymmetry of the PFR-axoneme interface, local arrangement of A and B tubules of DMTs, and orientation of axonemal dyneins, allowed unambiguous identification of the nine DMTs, numbered according to established convention^56^ (Fig. 1c).

Based on cross-sectional views in traditional transmission electron microscopy (TEM) studies, the PFR consists of three structurally distinct zones: proximal, intermediate, and distal^30^, and these are evident in tomograms (Fig. 1b, c; Supplementary Movie S1). One can readily identify repeating densities in the PFR distal zone that have the appearance of “comb teeth” in longitudinal sectional views (Fig. 1b, d; cyan arrows). Periodicities of major axoneme substructures, e.g., radial spokes (RS), outer arm dyneins (OADs) or inner arm dyneins (IADs), CPC and microtubule inner proteins (MIPs), vary but all are integer multiples of the underlying tubulin dimer repeat of 8 nm^26,57^. By contrast, we determined the periodicity of PFR distal zone repeating units to be 54 nm along the axoneme axis (Materials and methods). A 54 nm repeat interval is consistent with earlier measurements of 54−57 nm^44,48–50^. Since 54 is not a multiple of 8, resolving the details of axoneme and PFR structures simultaneously by sub-tomographic averaging is not possible. We therefore had to design a stepwise workflow incorporating both interactive and automatic particle-picking strategies (Materials and methods) to obtain sub-tomographic averaged structures for individual flagellum components, and then fitted them into a montage (Fig. 1d, e; Supplementary Movie S2) (Materials and methods). Visualizing the RS, tubulin dimer, OAD, CPC, PACs, and different zones of PFR in this way allows us to decipher interactions among these components, as detailed below.

### The PFR distal zone consists of a series of parallel SSN planes, aligned at 45° to the axoneme axis and interconnected by coiled-coil wires

**Fig. 2:**
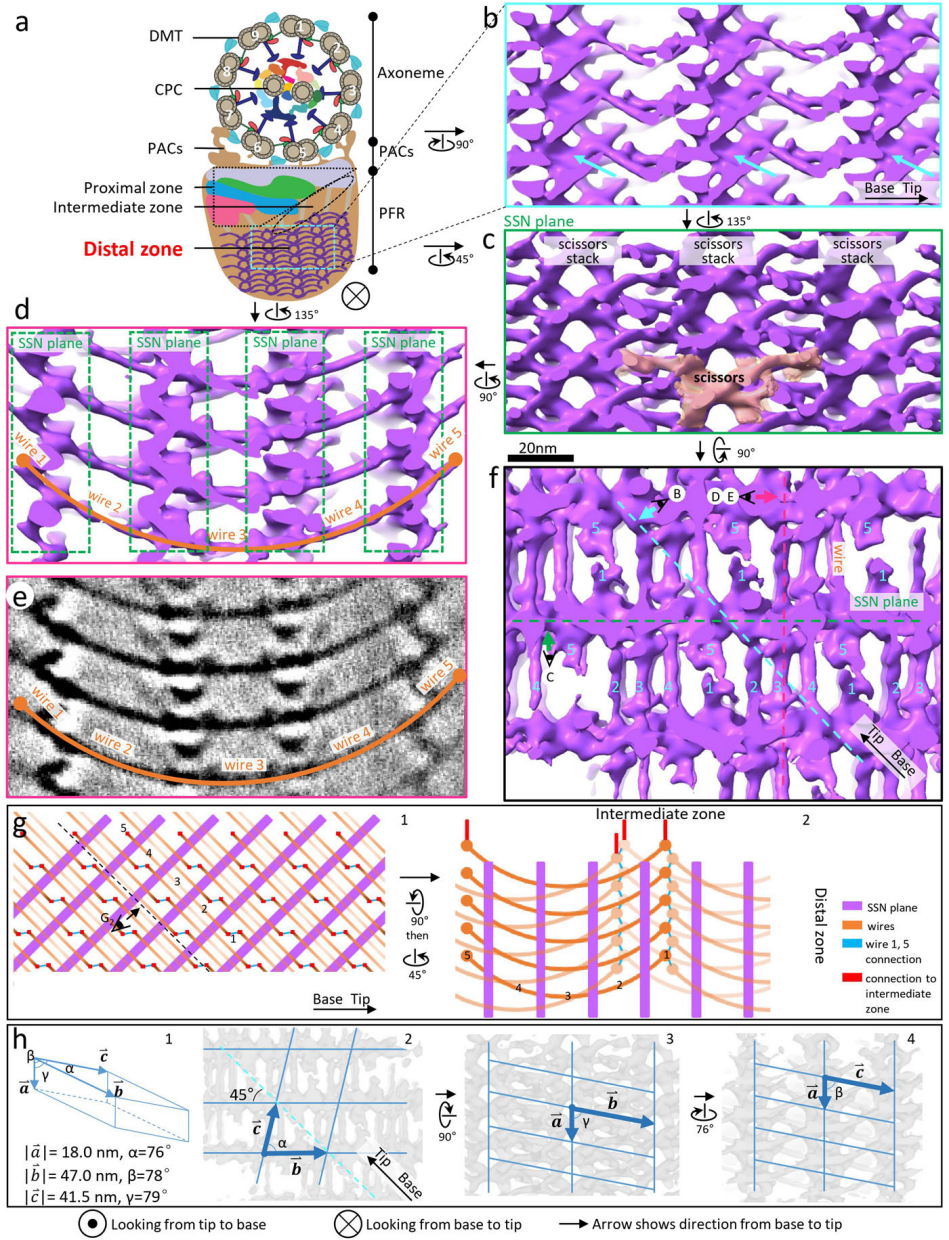
Structure of the paracrystalline PFR distal zone. **a** A schematic depicting a cross-section of *T. brucei* flagellum viewed from base to tip. **b**–**d**, **f** Shaded surface views of the boxed region in **a** after rotating as indicated. The “comb teeth” densities in Fig. 1b are resolved in greater detail in **b** and pointed out by cyan arrows in both. **b** is the side view; **c** is looking perpendicular to an SSN plane; **d** is looking parallel to the SSN planes; **f** is looking from the axoneme to the PFR distal zone. **e** Same view as **d**, showing density slice (5 nm thick). In **d** and **e**, wires 1–5 joining four consecutive SSN planes form a shape of a smiling face. The viewing directions of **b**–**e** are indicated within panel **f**, with letters corresponding to each panel and viewing directions indicated by an arrow next to the eye symbol. Scale bar for panels **b**–**f** is shown between panels **c** and **f**. **g** Schematics depicting arrangement of SSN planes (purple) and wires (orange) at the interface of the PFR distal and intermediate zones. The viewing direction of g_1_ is the same as that of **f** except that the horizontal direction is now along the axoneme axis. Decreased intensity of orange wires indicates further away from the viewer. **h** Geometry schematic illustrating the three vectors of the triclinic crystalline lattice discovered in the distal zone. The semi-transparent gray densities in the background of h_2_ is the same as that in **f**, and rotations for h_3_ and h_4_ are indicated.

Previous electron tomography studies have provided important insights into building blocks of the PFR^48–50^, but were unable to fully resolve the PFR organization. Using our newly developed script called *Propagate* (Materials and methods), together with the *PEET* program^55,58^, we were able to iteratively identify and refine the paracrystalline lattice parameters of the distal zone, first in one dimension along the axoneme axis and then in all three dimensions (see details in Materials and methods) (Fig. 2). Sub-tomographic averaging of the distal zone using 1362 sub-tomograms from 12 tomograms yielded an averaged 3D structure of the distal zone at ~28.5 Å resolution based on Fourier shell correlation (FSC) analysis at the 0.143 coefficient criterion (Supplementary Fig. S4a).

The refined paracrystalline lattice is triclinic (i.e., none of the three lattice angels is orthogonal) with the unit lengths of vectors $\mathop{a}\limits^{\rightharpoonup} ,\mathop{b}\limits^{\rightharpoonup} ,\mathop{c}\limits^{\rightharpoonup}$ of 18.0 nm, 47.0 nm, and 41.5 nm and angles of *α*, *β*, *γ* at 76°, 78°, and 79°, respectively (Fig. 2h). The dimension $\mathop{a}\limits^{\rightharpoonup}$ is along the vertical direction in Fig. 2a–e and 2h_1, 3, 4_; that is, $\mathop{a}\limits^{\rightharpoonup}$ is perpendicular to the axoneme axis. Surprisingly, the other two axes of the unit cell are neither along the axoneme axis (dashed cyan line, Fig. 2h_2_) nor orthogonal to dimension $\mathop{a}\limits^{\rightharpoonup}$. Thus, the unit cell is neither orthorhombic^48^, nor helical^44^. Rather, when examining from the top of the axoneme down to the PFR (Fig. 2f, h_2_), dimension $\mathop{b}\limits^{\rightharpoonup}$ is oriented approximately 45° from the axoneme axis, which may account for the ~45° angle previously observed in negative-stained samples^44,49^. Dimension $\mathop{c}\limits^{\rightharpoonup}$ is oblique (76°) to dimension $\mathop{b}\limits^{\rightharpoonup}$. The main building block of this plane is what we call density scissors (delineated by the tan surface rendering in Fig. 2c), which is visible when viewing the face of the plane from the perpendicular perspective. This scissors-like building block differs from a previously proposed “jackscrew” model, which was based on viewing the distal zone from the axoneme side of the PFR^50^. Density scissors stack vertically upon one another along dimension $\mathop{a}\limits^{\rightharpoonup}$ (Fig. 2c, h_3_), with 4−6 scissors per stack. Each stack in turn connects horizontally with adjacent scissors stacks, forming a planar network of stacks, which we term a “scissors stack network” plane (SSN plane), that is oriented 45° to the axoneme axis. Each SSN plane encompasses 5 stacks, 1 with 5 pairs of scissors, 3 with 6 pairs of scissors, and one near the boundary of the PFR on DMT4 side with 4 pairs of scissors but with densities exhibiting differences from the other 4 stacks. Thus, a 20 µm long PFR would include ~370 SSN planes with ~27 pairs of scissors per plane.

Extending between SSN planes are thin densities, which we call wires. When viewed parallel to SSN planes (Fig. 2d, e; Supplementary Movie S3), wires from adjacent planes appear to extend contiguously to form a smile-like arc (0.8 radians) that spans four SSN planes, with two end segments (wires 1 and 5) and three middle segments (wires 2–4). When viewed from above (Fig. 2f), the end segments of each arc deviate slightly from a straight line formed by the three middle segments. Wire 1 from one arc abuts wire 5 of an adjacent arc and their structures appear distinct (Fig. 2f). When the density threshold is lowered, wires 1 and 5 appear to interact (Fig. 2f, g; Supplementary Movie S3). In addition, near the interface of the distal and intermediate zones, wires 1 and 5 extend to make direct contact with densities in the intermediate zone (Fig. 2g; Supplementary Movie S4; see also Fig. 4b, c). Therefore, in the structure examined, the distal zone is a remarkable and intricate 3D nanoscale crystal consisting of many SSN planes aligned 45° to the axoneme axis and joined together by density wires, with ~90% empty space when calculated using a threshold as in Fig. 2f.

**Fig. 3:**
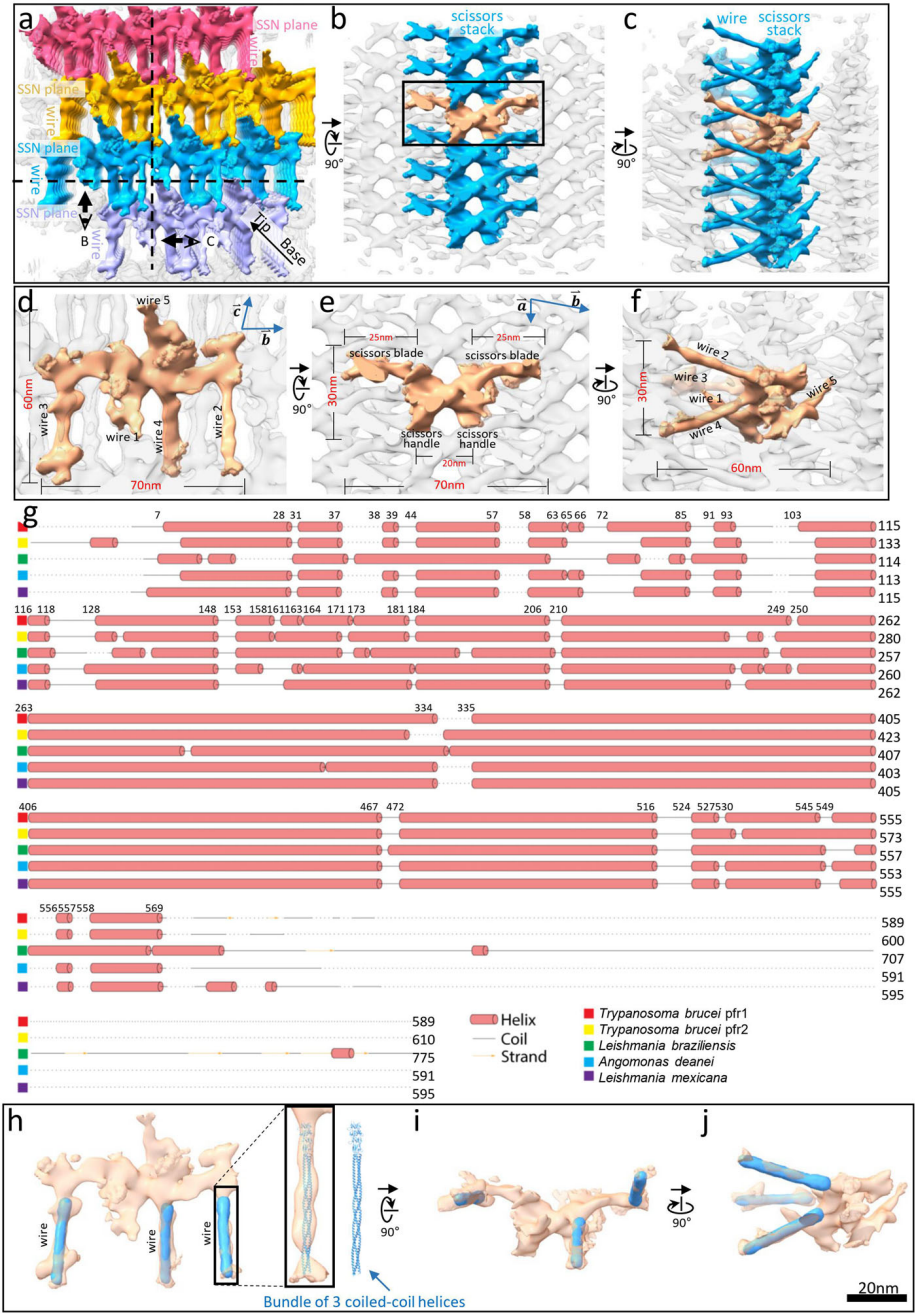
Paracrystalline network of the PFR distal zone and structural interpretation of subunits. **a** An overall surface view of a PFR distal zone encompassing four SSN planes (different colors) and their connecting wires, showing the paracrystalline arrangement. The view is looking at the PFR distal zone from the axoneme. **b**, **c** Two orthogonal surface views of **a** at the sectional planes indicated by the dashed lines in **a**, showing a single scissors density with wires (tan) in a stack (blue) and joined to adjacent scissors stacks (gray), forming an SSN plane of the paracrystalline zone. **d**–**f** Three orthogonal zoom-in views of a single scissors density with wires (tan) within the paracrystalline distal zone (gray). Viewing directions in **d**, **e**, and **f** are the same as in **a**, **b**, and **c**, respectively. **g** Predicted secondary structures of major PFR proteins from the indicated organisms, shown according to their sequence alignment. Amino acid residue numbers of *T. brucei* PFR1 are indicated on the top. **h**–**j** Three orthogonal views of a single scissors density with wires (transparent tan) showing a 3 coiled-coil helix bundle (PDB: 6GAO)^103^ (blue) fitted into the wires.

To interpret structures constituting the unit cell of the distal zone, we examined elements that make up each SSN plane (same color in Fig. 3a) and connect with neighboring SSN planes (different colors in Fig. 3a). We segmented these elements in such a way that each scissors density and its connecting wires remain together (Fig. 3b, c). These structural elements can be brought together within a single unit cell by translation of one unit length along the vectors of the triclinic unit cell (Fig. 3d−f). Figure 3b is the view looking perpendicularly at the SSN plane and highlights one scissors stack in blue, with a single pair of scissors density colored tan. The handles of the scissors are at the bottom with curved blades projecting toward the top (Fig. 3b, e). Rotating this view 90 degrees (Fig. 3c) reveals the wire densities that extend between adjacent SSN planes described above. Figure 3d−f shows three orthogonal views of the structural elements that comprise a single unit cell, with dimensions of the unit cell (as defined in Fig. 2h) indicated with vectors and wires 1−5 labeled.

Current resolution of the PFR structure precludes identification of molecules that make up the scissors densities and wires, but the extended rod-shape of wires is consistent with structures exhibited by coiled-coil helices. Biochemical analyses indicate that major structural components of the PFR are two proteins, PFR1 and PFR2^59,60^. Deletion mutants of either PFR1 or PFR2 failed to assemble a complete PFR structure^21,32,61,62^, demonstrating that, despite sharing high-percentage amino acid identity, PFR1 and PFR2 are essential and non-redundant components of the PFR. In some cases, a rudimentary proximal domain is retained^21,32,62^. Secondary structure predictions of PFR1 and PFR2 from *T. brucei* and homologs from other *kinetoplastids* showed that more than 80% of the protein is predicted to form helices (Fig. 3g)^60^. The N-terminal half contains helices of short lengths, followed by unusually long helices. The longest predicted helices of PFR1 and PFR2 contain 258 amino acids and 222 amino acids, respectively, which correspond to a length of 41.3 nm and 35.5 nm, respectively (~1.6 Å/amino acid in an alpha helix). In our sub-tomographic averaged structure, the lengths of individual rod-shaped density wires range from 40 nm to 45 nm. The diameter of the wire, ~4 nm, would accommodate a coiled coil of 3−4 helices (Fig. 3h−j), suggesting that multiple subunits of PFR1 and PFR2 molecules could contribute to each arm of the wire. The shorter helices and coiled-coil sequences predicted could contribute to the formation of the globular region of the scissors densities. Additional PFR components have been identified^42^, including PFC3 and PAR1 that are predicted to assemble into extended coiled-coil structures, and we expect these may also contribute to wires or other PFR structures. However, unlike PFR1 and 2, RNAi knockdown of PFC3 or PAR1 does not noticeably affect PFR ultrastructure^63^, indicating they are not required for assembly of the main PFR structural elements.

### Contiguous overlapping layers in the proximal zone and flexible linkages in the intermediate zone

**Fig. 4:**
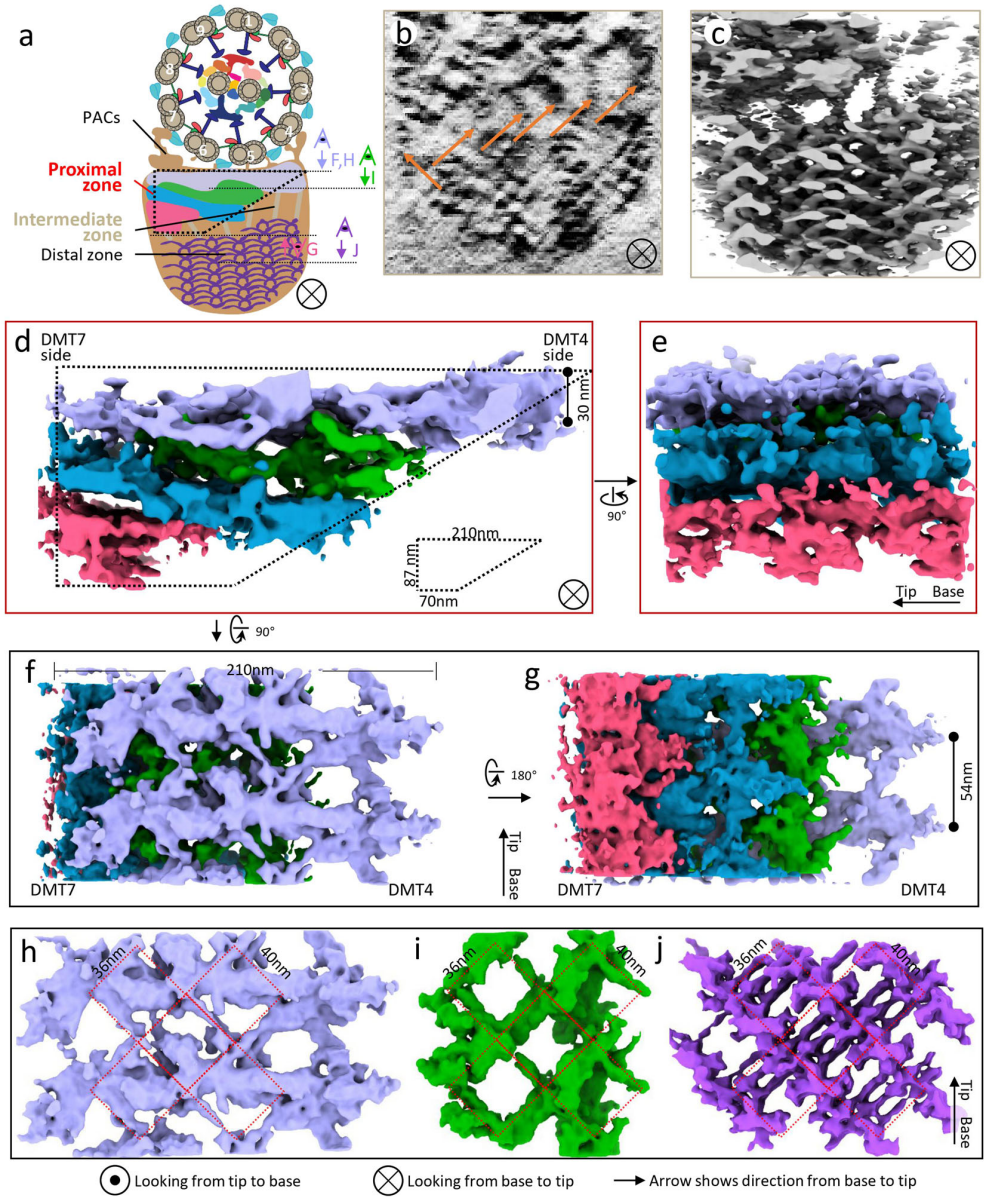
Structure of the proximal zone and its structural continuity with the intermediate and distal zones. **a** A schematic illustrating the location of the trapezoid-shaped proximal zone with respect to the rest of the flagellum. **b**, **c** Cross-section density slice (10 nm thickness) from a single tomogram (**b**) and the surface view of the same region after sub-tomographic averaging displayed at a low-density threshold (**c**). The connecting densities of the intermediate zone (arrows in **b**) contact wires of the distal zone. **d**–**g** Cross-section (**d**), side (**e**), top (**f**), and bottom (**g**) surface views of the trapezoid-shaped proximal zone after sub-tomographic averaging. The four density layers distinguishable by gaps are colored differently. Viewing angles are shown in **a**. **h**–**j** Top views of the light purple (**h**) and green (**i**) proximal zone layers, alongside the same view of the distal zone (**j**), highlighting the contiguous 36 by 40 nm lattice pattern (red) in all three.

The organizations of the PFR proximal and intermediate zones remain a mystery, likely due to difficulties in sub-tomographic averaging caused by lack of knowledge about the periodicity, flexible nature of the structure, and the large volume to resolve. In 3D reconstructed tomograms after missing-wedge compensation, one can readily discern multiple densities in the intermediate zone, consistent with TEM from thin cross-section of embedded trypanosome flagella^49^ (Fig. 4a, b). These densities connect to wires 1 and 5 of the distal zone (Fig. 4b, orange arrows), providing a direct link between structurally distinct PFR zones. Sub-tomographic averaging did not improve resolution of these densities (Fig. 4c), suggesting that they might be flexible or present polymorphic features.

In the sub-tomographic average of the proximal zone, the overall cross-section contour approximates a right-angle trapezoid with the right-angle side corresponding to the DMT7 side of the axoneme (Fig. 4d). The approximate dimensions of this trapezoid are base lengths of 210 nm and 70 nm, and height of 87 nm. Unlike the clearly resolved density elements in the distal zone described above, proximal zone density elements are convoluted and hard to distinguish from one another. Nonetheless, we were able to segment these densities into four layers when viewed in cross section, by following the gaps visible as shown in Fig. 4d. Three of these layers are readily visible in longitudinal views from outside the PFR (pink, blue, and light purple in Fig. 4e; Supplementary Movie S5). When viewed from the axoneme looking toward the PFR, the light purple layer is dominated by an elongated density extending perpendicular to the PFR axis and spanning from DMT7 to DMT4 of the axoneme. This density measures ~210 nm long and ~40 nm wide, with thickness up to 30 nm on the DMT4 side. This density repeats along the axoneme axis every 54 nm (Fig. 4f, g), which is the same interval observed for the distal zone repeat. The other proximal zone layers (pink, blue, green) are best visualized when viewed from the PFR distal zone looking toward the axoneme, which allows the visualization of all four layers of the PFR proximal zone (Fig. 4g). Diagonal densities join adjacent repeats within each layer and form interconnections between layers (Fig. 4h, i; Supplementary Movie S5). These densities form lattice-like networks, when examined from the axoneme interface looking toward the PFR (Fig. 4h, i; Supplementary Movie S5). Though different from one another, these networks and that in the distal zone (Fig. 4j) appear to be congruent with the rectangular, 36 nm by 40 nm repeating units, observed in the green layer of proximal zone (Fig. 4j). This congruence suggests presence of organizational linkages extending from the distal zone into the intermediate and proximal zones, and a mechanism for mediating continuity between the linear repeat of the axoneme with the diagonal repeat of the PFR distal zone.

### PAC structures bridge different repeats of the PFR and axoneme

**Fig. 5:**
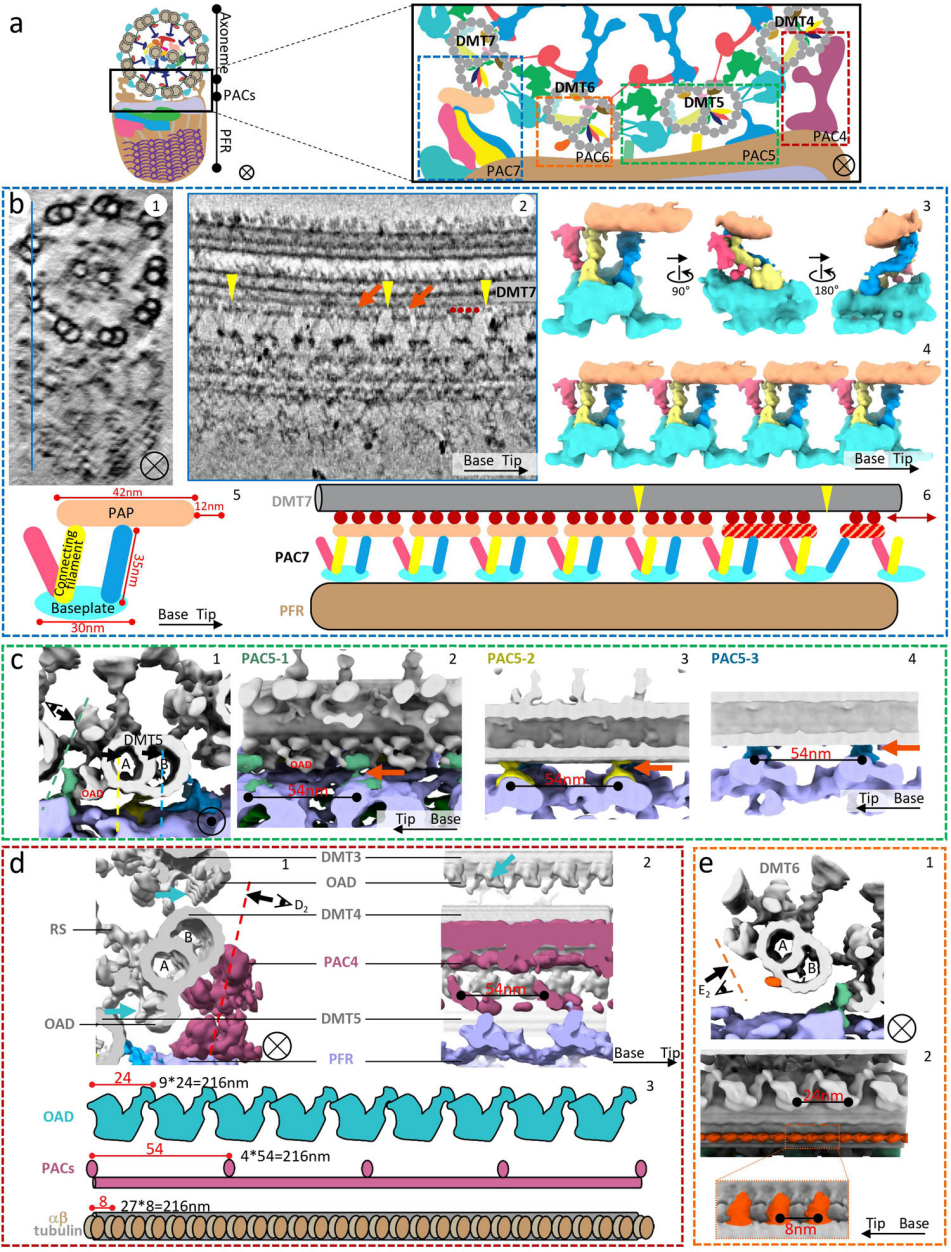
Structures of PFR-axoneme connectors (PACs). **a** A schematic with an enlarged inset illustrating the locations of the four PACs in the *T. brucei* flagellum. The colored boxes enclosing each PAC approximate the regions used for obtaining sub-tomographic averages detailed in **b**–**e**. **b** Details and dynamics of the PAC7 structure. A 10nm-thick longitudinal density slice (b_2_) from the location indicated by the blue line in B_1_ shows that PAPs have variable sizes (orange arrows), are connected to DMT7 by globular densities (red dots) and are separated by variable gaps (yellow arrowheads). Three surface views of the averaged PAC7 structure (b_3_) show its PAP, baseplate, and connecting filaments. Four PAC7 structures are montaged (b_4_) according to the locations in b_2_ to illustrate connectivity. Schematics in b_5_ and b_6_ depict dimensions of PAC7 components (b_5_), illustrate how PAPs interact with DMT7 through globular densities (red dots) (b_6_), and that sliding (double-headed arrow) might produce the heterogeneity in size and spacing observed for PAPs (b_6_). **c** Zoomed in surface views of the PAC5 region showing details and interconnections. Each panel is a montage of five sub-tomographic averages: the 96 nm averaged axoneme (gray), 54 nm averaged PFR proximal zone (light purple), PAC5-1 (green), 5-2 (yellow), and 5-3 (blue). **d** Two orthogonal zoomed in surface views (d_1_ and d_2_) and schematics (d_3_) of the PAC4 region, illustrating periodicities of OAD, PACs, and the α/β-tubulin dimer. Panels d_1_ and d_2_ are each a montage of three sub-tomographic averages: the 96 nm averaged axoneme (gray) and the 54 nm averaged PFR proximal zone (light purple) and PAC4 (dark purple). **e** Zoomed in surface views of the DMT6 region showing details. Panel e1 is a montage of four sub-tomographic averages: the 96 nm averaged axoneme (gray), the 8 nm averaged DMT6-MOP-B1,2 (orange), and the 54 nm averaged PFR proximal zone (light purple) and PAC5-1 (green). Panel e_2_ is a montage of two sub-tomographic averages: the 96 nm averaged axoneme and the 8 nm averaged DMT6-MOP-B1,2 (orange).

A central question about *T. brucei* flagellum biology concerns the mechanism of PFR attachment to the axoneme at DMT4-7. Our finding that the PFR and DMT repeating unit dimensions are mismatched, 54 vs 8 nm, makes this a particularly challenging problem. Filaments connecting the PFR to DMT7 have been described^47,49,64^, but the 3D arrangement and structural details were limited and connections to DMT4, 5, and 6 are almost completely uncharacterized. We therefore performed sub-tomographic averaging of local volumes at the PFR-axoneme interface to resolve individual structures of connections at DMT4-7, which we term PFR-axoneme connectors (PACs) (Fig. 5a).

Prior studies describe PAC7 as filamentous connections between the PFR and DMT7^47,65^. Our structure reveals PAC7 is actually comprised of four components (Fig. 5b2-b5): a PFR-proximal baseplate (~30 nm in diameter), a peri-axonemal plate (PAP, approximately 42 by 20 by 12 nm in size), filaments (~35 nm in length) connecting the baseplate and PAP, and small globular densities that connect the PAP to the microtubule lattice of the DMT7 B-tubule at protofilaments B2-4 (Supplementary Fig. S3). There are typically three connecting filaments per baseplate, with two parallel filaments connecting to one PAP and the third extending from the baseplate to contact the PAP of an adjacent PAC7. Positioning of connecting filaments along the PFR-axoneme interface is not entirely uniform (Fig. 5b2, b6) and structural details of these filaments become smeared in the sub-tomographic average of PAC7 (Fig. 5b3, b4). The PAC7 baseplate exhibits a periodicity of 54 nm, consistent with the PFR repeating unit. However, the PAP does not repeat in an entirely regular fashion (Fig. 5b2, b6), likely reflecting the need to interface the different repeats of the PFR (54 nm) and axoneme (96 nm). Thus, our cryoET structure resolves the structural basis for bridging the distinct repeating unit dimensions of two megastructures, the PFR and axoneme, which must act together to support unique motility of the trypanosome cell.

PAC5 includes three separate connections: PAC5-1, PAC5-2, and PAC5-3 (Fig. 5c; Supplementary Fig. S3d, h, l), which connect to OAD, and protofilaments B1, 2 and B5 of DMT5, respectively (Fig. 5c1; Supplementary Fig. S3). These results support an earlier report that PFR may influence motility through direct interaction with dynein^49^. In longitudinal views, PAC5-1, 5-2, and 5-3 each include two densities spaced 54 nm apart.

PAC4, when viewed in cross-section, is a large globular density that forms a U-like structure at the axoneme, making separate contacts with the A and B-tubules of DMT4 at protofilaments A9, 10 and B1, 3 (Fig. 5d1; Supplementary Fig. S3). PAC4 has a periodicity of 54 nm along the flagellum (Fig. 5d2), consistent with the repeating unit of the PFR. Because 216 is the least common multiple of 8, 24, and 54, a structural unit encompassing the PFR and its contact points on the axoneme, α/β-tubulin dimer, and OAD, would repeat each 216 nm (Fig. 5d3), while a unit that also includes the 96 nm repeat of RS-IAD-NDRC would repeat each 864 nm.

We did not observe obvious densities for PAC6 in the PFR sub-tomographic average. However, in the axoneme sub-tomographic average we did resolve a novel “microtubule outer protein” (MOP) on DMT6 (orange-colored in Fig. 5e), which we term DMT6-MOPB-1,2, because it is attached to the DMT6 B-tubule protofilaments 1 and 2 (Fig. 5e; Supplementary Fig. S3). DMT6-MOPB-1,2 has a periodicity of 8 nm, consistent with the periodicity of the α/β-tubulin dimer.

### Structure of the *T. brucei* central pair complex

**Fig. 6:**
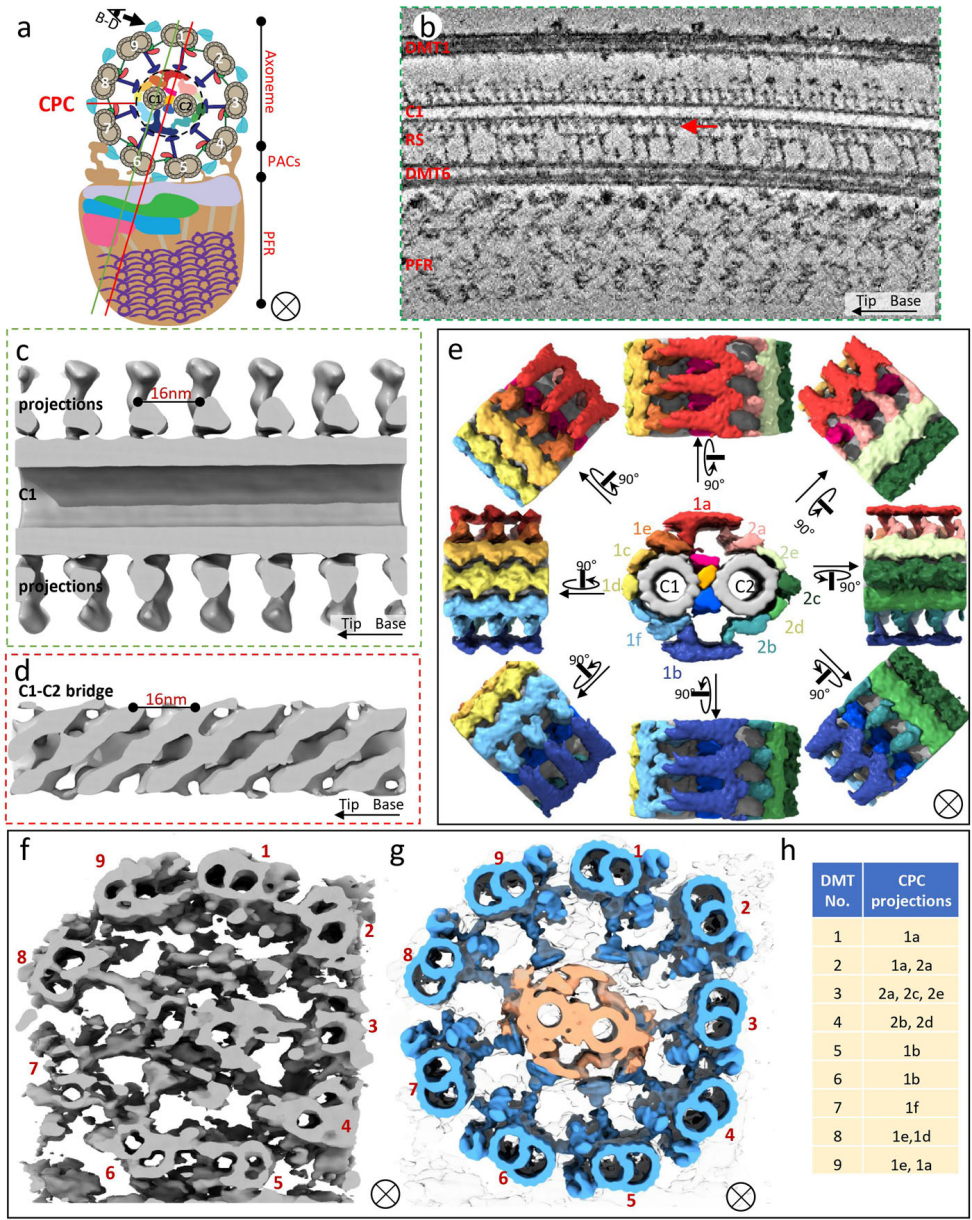
Structure of the *T. brucei* central pair complex (CPC). **a** A schematic illustrating the location and relative orientation of the CPC with respect to the surrounding 9 DMTs. The section planes are indicated for panels **b** and **c** (green line), and panel **d** (red line). **b** A 10nm-thick density slice through a tomogram along the plane marked by the green line in **a**. Connections between the RS and CPC are visible (red arrow). **c** Surface view of the CPC 96 nm sub-tomographic average sectioned along plane marked by the green line in **a**, showing C1 projections, which repeat at 16 nm intervals. **d** Surface view of the CPC 96 nm sub-tomographic average sectioned along the plane marked by the red line in **a**, showing the bridge structure between the C1 and C2 microtubules of CPC, which repeats at 16 nm intervals. **e** Cross sectional (middle) and rotational (surrounding) surface views of the CPC 16 nm sub-tomographic average, showing the 1a–f and 2a–e projections, C1 and C2 microtubules (gray), and tripartite bridge structure (pink, light orange and blue). **f**–**g** Connections between CPC and RS. Surface view of a single tomogram (**f**), fitting (**g**) of 96 nm averaged CPC and individual DMT-RS structures within the tomogram shown in **f**. **h** Summary of contacts observed between CPC and RS from each DMT (based on Fig. 6g; Supplementary Fig. S2).

The CPC (Fig. 6a) is an essential regulator of axoneme motility, functioning with the RS to transmit mechanochemical signals across the axoneme^27,54,66^. Importance of CPC function is evidenced by several human diseases associated with CPC abnormalities^67^ and a requirement of CPC proteins for *T. brucei* motility^34,68^. We therefore determined the 3D structure of the *T. brucei* CPC in situ. The CPC is visible in individual tomograms (Figs. 1b, 6b; Supplementary Fig. S2). Sub-tomographic averaging at 96 nm periodicity shows densities protruding outward from the central pair microtubules (Fig. 6c) and diagonally arranged densities of the tripartite bridge^69^ between C1 and C2 microtubules (Fig. 6d). Major densities repeated at an interval of 16 nm (Fig. 6c, d). We therefore did sub-tomographic averaging at 16 nm, yielding a 25 Å resolution sub-tomographic average structure (Fig. 6e; Supplementary Fig. S4b and Supplementary Movie S6), in which we resolved the C1 and C2 microtubules, as well as densities corresponding to 11 projections and tripartite bridge between C1 and C2 described for the *Chlamydomonas* and *Strongylocentrotus* CPC^69^ (Fig. 6e). Interfacing with RS is critical for CPC function^24,25,27^ and extensive contacts between RS and CPC projections are observed in the *T. brucei* structure (Fig. 6b, f–h; Supplementary Fig. S2). These extensive contacts may contribute to restricted orientations of the CPC relative to the DMTs in *T. brucei*^34,68,70^. Our results provide the first 3D structure for the CPC in trypanosomes, revealing overall conserved features and extensive direct contacts with the RS.

## Discussion

In this study, we have used a combination of cryoET, machine learning-based missing wedge compensation, and sub-tomographic averaging to resolve the previously unknown molecular structures of the PFR, CPC, and PACs in *T. brucei*. The structures reported here provide insights into flagellar motility and mechanical bionics.

To date, studies of structural mechanisms underpinning flagellum beating have focused almost exclusively on organisms with planar axoneme beating, and have mostly ignored extra-axonemal filaments^23^. However, helical waves are common among microbes, including important pathogens^5,8,36,71^, and even occur in human sperm^72^. Non-planar helical motion may contribute to microbial pathogenesis^12^, as it is recognized to facilitate propulsion through viscous environments^73^, such as host tissues. Meanwhile, extra-axonemal filaments are common features of eukaryotic flagella^16,17,19^. Therefore, a full understanding of biomechanics of cell propulsion requires structural analysis of flagella from organisms that support helical motion, such as *T. brucei*, and requires analysis of extra-axonemal structures, such as the PFR.

**Fig. 7:**
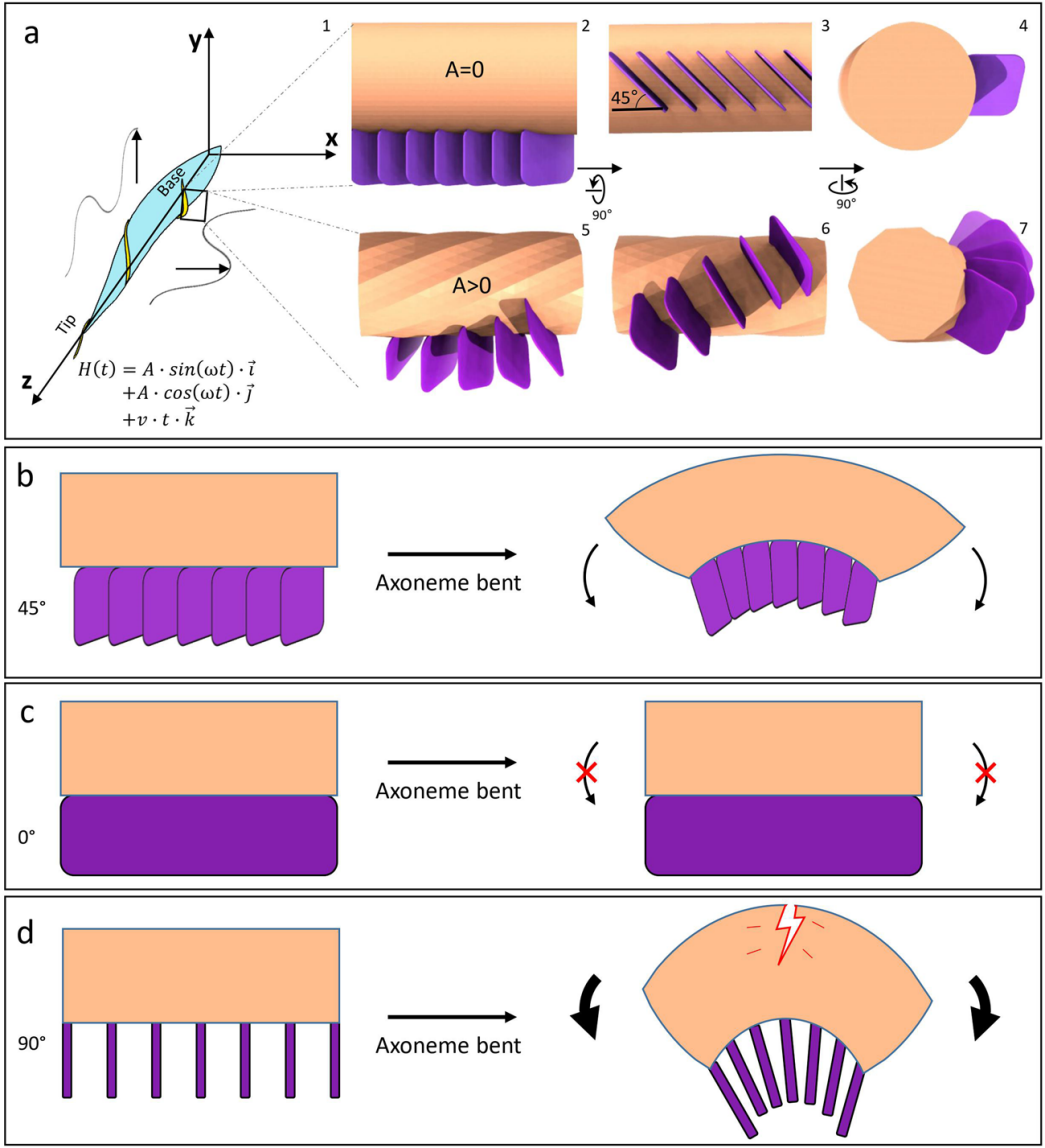
Model for SSN planes supporting non-planar helical wave of the *T. brucei* flagellum. **a** Schematics depicting movement of the *T. brucei* cell with the flagellum’s non-planar helical wave decomposed into *x*, *y*, and *z* components (left, a_1_). $\vec i,\vec j,\vec k$ are unit vectors along *x*, *y*, and *z* axis, respectively. The right panels (a_2–7_) show the architectural relationship between the axoneme (tan) and SSN planes of the PFR (purple) on the flagellum in the resting (amplitude *A* = 0) and beating (*A* > 0) states. Note that non-planar helical wave would introduce local twist to the flagellum (a_5–7_). **b**–**d** Three different axoneme bending scenarios with SSN planes placed at either 45° (observed, **b**), or hypothetically, 0° (**c**) or 90° (**d**), with respect to the axoneme axis.

For the sake of illustration, helical waves can be decomposed into *x* and *y* oscillations each described by a sinusoidal function $H(t) = A \cdot {\mathrm{sin}}\left( {\omega t} \right) \cdot \vec i + A \cdot {\mathrm{cos}}\left( {\omega t} \right) \cdot \vec j + v \cdot t \cdot \vec k$ where $A$,$\omega$, $v$ are amplitude, rotational speed and forward speed, respectively, and $\vec i,\vec j,\vec k$ are orthogonal unit vectors in the *x*, *y*, and *z* axis, respectively (Fig. 7a). In addition, since points along the axoneme filament are connected, we must also consider twist introduced by helical waves *(*i.e., the situation for *A* > 0 in Fig. 7a). Therefore, the PFR of *T. brucei* must provide elastic bending resistance^21^ while being flexible enough to support the axoneme as it executes complex helical motion^5,36,74,75^. SSN planes placed along the axoneme at about 45° to the axoneme axis offers an excellent solution to these competing needs (Fig. 7b). We propose that the two-dimensional SSN planes provide a rigid component for support and resistance, while connection of these planes in the third dimension by coiled-coil helix bundles (i.e., wires in Fig. 3) provides elasticity. Such organization imparts integrity to the axoneme, yet still allows sinusoidal oscillation in both *x* and *y* directions and the 45° orientation of SSN planes also provides elastic resistance and support both across and along axoneme DMTs. By contrast, placing SSN planes at 0° or 90° with respect to the axis of axoneme would prohibit oscillation in the *y* direction (Fig. 7c), or lend less support along axoneme DMTs (Fig. 7d), respectively. A 45° orientation may also contribute to the helical bending of the axoneme.

A distal zone comprised of SSN planes interconnected by wire-like densities differs from a previously proposed “jackscrew” model for the PFR distal zone^50^. The jackscrew model was based on viewing the distal zone from the axoneme toward the PFR, which revealed two sets of linear densities intersecting diagonally to form a lattice of diamond shapes (Fig. 3b, d, f in^48^). Viewing from this perspective, we also observe a lattice-like arrangement of diagonally intersecting densities (Fig. 4j). However, as noted previously^48^, there is no density connecting opposing vertices of each diamond shape, i.e., no screw for a jackscrew (Fig. 4j). Moreover, the earlier work did not resolve the structural units that comprise each diagonal density in the lattice, i.e., stacked scissors of SSN planes and wire densities that connect them as described here.

For the PFR to fulfill motility functions in unison with the axoneme, these two massive structures must be interconnected in a way that enables mechanochemical signals to be transmitted to all participating components. Such signals may go both ways, either from axoneme to PFR or vice versa, as the PFR is a platform for Ca++ and cAMP signaling systems^40,42^. The observed structural organization of connections within and between the axoneme and PFR is consistent with such needs. Within the axoneme, signaling between DMTs is supported by nexin links that connect adjacent DMTs^76^. Our prior studies^26^ demonstrate capacity for DMT-DMT signaling in *T. brucei*, by confirming the presence of nexin links, and identifying novel, lineage-specific connections between adjacent DMTs^26^. In the present study, our observation of direct interaction between RS and CPC (Fig. 6b, f, g; Supplementary Fig. S2), is likewise consistent with the role of the RS in transmitting signals through the CPC to DMTs across the axoneme reported in other organisms^27,54,66^. Extensive contact between RS and CPC may also contribute to limited rotation observed for the *T. brucei* CPC relative to DMTs^34,68,70^. Signals from the axoneme may in turn be transmitted directly to the PFR proximal zone through the PACs. Contiguous structural connections extending through the proximal, intermediate, and distal zones (Fig. 4b, c, h−j; Supplementary Movies S2, S4, S5) allow these signals to be propagated all the way to the distal zone, which provides a highly organized spring-like structure that can store and release mechanical energy.

Structural details of PFR connections to the axoneme provide insight into how the PFR may influence axoneme beating. The mismatch in periodicity of the axoneme (8 nm) and the PFR (54 nm) is reminiscent of symmetry mismatch often observed in structures involved in dynamic biological processes, such as those of the DNA translocation portals in viruses^77,78^. Dynamic interaction is also supported by the observed heterogeneity in spacing of the PAC7–DMT7 interface (Fig. 5b), as this heterogeneity in spacing suggests capacity for sliding of PAC7 on this side of the axoneme. In addition, PAC4 and 5 appear to have more regular longitudinal spacing, and such an arrangement could support or even amplify helical bending, as it would present more resistance to bending on the DMT4 versus DMT7 side of the axoneme. A mismatch in periodicity also suggests that, although PFR assembly is coordinated with and dependent on axoneme assembly^79,80^, PFR assembly is not templated by the axoneme. This interpretation is consistent with RNAi knockdown studies showing proteins that are not part of the PFR are nonetheless required for proper PFR assembly^81–83^, indicating that PFR assembly is a multistep, well-controlled process with assembly steps in the cytoplasm as well as flagellum. Finally, direct connection of PAC5-1 to dynein motors on DMT5 (Fig. 5c1, c2)^49^ will exert substantial influence on dynein orientation. Because reorientation of axonemal dynein during the beat cycle is a major mechanism of axonemal beat regulation^23,24,84^, PAC5-1 provides a mechanism for the PFR to directly control axonemal beating.

In summary, our studies provide a high-resolution 3D description of an extra-axonemal structure, giving insight into how these common yet enigmatic components of eukaryotic flagella contribute to axonemal beating. From the standpoint of bionics, with less than 10% space filled, the PFR of trypanosomes may provide an example of cellular tensegrity—biological entities that embody a fine balance between strength and flexibility, owing to opposing forces of compression or tension—and should inform future bioengineering and mechanical design of nanomachines and microswimmers^85,86^.

## Materials and methods

### Sample preparation and cryoET

*T. brucei* bloodstream form single marker (BSSM) cells^87^ were used, and details for culturing, flagella isolation, and cryoET were described previously^26^. Briefly, demembraned flagella were isolated and vitrified on quantifoil grids with 5 nm gold particles. With *SerialEM*^88^, tilt series were collected from straight segments near the center part of full-length flagella, spanning the middle third between the basal body and tip, in a Titan Krios instrument equipped with a VPP, a Gatan imaging filter (GIF) and a post-GIF K2 direct electron detector in electron-counting mode. Frames in each movie of the raw tilt series were aligned, drift-corrected, and averaged with *Motioncorr*^89^. The tilt series micrographs were aligned and reconstructed into 3D tomograms by either weighted back projections (WBP, for sub-tomographic averaging) or simultaneous iterative reconstruction technique (SIRT, for visualization and particle picking) using the *IMOD* software package^90^. The contrast transfer function (CTF) was determined by *ctffind4*^91^ and corrected with the *ctfphaseflip* program^92^ of *IMOD*. With phase plate, the CTF is insensitive to the sign of the defocus value being negative (under-focus) or positive (over-focus)^93^ so CTF of micrographs obtained with phase plate were approximated when CTF rings were not readily detected.

### Missing-wedge compensation

Demembranated *T. brucei* flagella samples typically lie on the cryoEM grids with a preferred orientation due to the presence of the PFR. To alleviate the missing-wedge problem associated with preferred orientation, we used a novel deep learning-based method developed to compensate for missing-wedge problem (Liu et al., unpublished program). Using tilt geometry and resulting tomograms as inputs, the program iteratively learns how to fill in missing information.

### Sub-tomographic averaging

In our sub-tomographic averaging scheme performed using *PEET*, each particle is a 3D sub-volume of the tomogram corresponding to the repeating unit of the component of interest, i.e., PFR distal zone, PFR proximal zones, PACs, or CPC.

#### PFR distal zone

For the PFR distal zone, sub-tomographic averaging required first defining the repeating unit through multiple rounds of *PEET* trials, and then using this defined repeating unit to re-pick particles automatically for the final round of *PEET* refinement, leading to a final sub-tomographic average at the best possible resolution.

To define the repeating unit a priori, we first performed crude sub-tomographic averaging with 4x binned (resulting a pixel size of 10.2 Å) SIRT tomograms. Initially, we manually picked particles (190 × 190 × 190 pixels) by taking advantage of the repeating comb teeth visible in the PFR distal zone in the raw tomograms (e.g., Figs. 1b and 2b, cyan arrows). For each particle, this process records the *x*, *y*, *z* coordinates of two points in the tomogram, one at each end of a tooth. The coordinates for all picked particles in each tomogram were saved into a coordinates model.mod file. With this model file as the input, we then ran *stalkInit* of the *PEET* package to generate three output files—motive list.csv file (translation and rotation parameters), coordinates model.mod file (central coordinates of particles) and rotation axes.csv file (vectors representing rotation axes for all particles)—for each tomogram. All these picked particles were summed together to generate a featureless volume, which was used as the initial reference of the first cycle of *PEET*. We then ran *PEET* iteratively by gradually decreasing the search range for both angular and distance parameters. For the angular search range, the parameters decreased from “180° max with 60° step in *Phi* (y axis), and 9° max with 3° step in both *Theta* (*z* axis) and *Psi* (*x* axis)”, to “3° max with 1° step in all *Phi* (*y* axis), *Theta* (*z* axis), and *Psi* (*x* axis)”. For the distance search range, the parameters decreased from 10 pixels to 2 pixels along all three axes. In addition to decreasing the search ranges, the reference was also updated by using the result of the previous round of *PEET* sub-tomographic averaging. This process was iterated until the averaged structure converged, and no improvement in the averaged structure could be observed. In this converged average, repeating densities, characteristic of those ultimately resolved in the lattice in Fig. 4j, were observed.

The repeating unit parameters defined above were utilized to re-pick particles following the 3D lattice. To do this for many tomograms automatically, we developed a bash script, *propagate.sh*, and used it to pick particles encompassing all repeating units within each tomogram, taking advantage of the unit cell dimensions of the above-observed lattice in all three orthogonal directions. At this stage, the sub-tomographic average volume resulting from the above initial *PEET* process contains more than one repeating unit. Though our script allows multiple repeating units in three dimensions, in the current case, we only needed to pick up all repeating units in the two dimensions, i.e., within an SSN plane, because the above manually picked particles cover only one unit-cell length along axoneme axis. Using the 3D visualization tools of *IMOD*, we identified 22 repeating units within each SSN plane. We carefully measured the *x*, *y*, and *z* distances between the center position of the current average volume and the center of each of the 22 repeating units and list each set of *x*, *y*, *z* distances in separate lines in a propagation list file. The script *propagate.sh* takes as inputs the follwoing: the propagation list file, and a set of three alignment files—including a motive list file (translation and rotation parameters), a coordinates model file (central coordinates of particles), and a rotation axes file (vectors representing rotation axes for all particles) for each tomogram—generated from the above *PEET* round. The output includes a single *prm* file (file pathway information) for the entire project and a new set of three alignment files for each tomogram to be used as the input files for the next round of *PEET*. This re-picking process ended up with 22 times as many particles as in the initial manual picking process.

With these re-picked particles, one round of iterative *PEET* refinement was performed by loading the new alignment and *prm* files to generate the final sub-tomographic average for the PFR distal zone. As before, the search ranges were gradually decreased and the average result from each prior refinement cycle was used as the updated reference for the subsequent cycle of refinement. The refinement was terminated when the averaged structure converged, and no further improvement could be seen. From the *unMasked* averaged density map file automatically generated by PEET during the refinement, the repeating length along the axoneme axis was measured to be 54 nm. Unit cell dimensions were also measured as reported as the final parameters in Fig. 2h.

#### PFR proximal and intermediate zones, and PACs

For sub-tomographic averaging of PFR proximal and intermediate zones, and PACs, the method is the same as that of the PFR distal zone, except that there was no need to run *propagate* because the repeating unit is only one dimensional. Instead, we identified prominent repeated densities from raw tomograms to be used for manual particle picking. For example, we used the baseplate density attached with 3 filaments (Fig. 5b) for manual picking PAC7. After iterative sub-tomographic averaging for the component of interest, the *unMasked* averaged density map file automatically generated by *PEET* was used to measure the dimension of a repeating unit.

#### CPC

To identify the periodicity of CPC, we first evaluated the tomograms and did a trial of sub-tomographic averaging using 96 nm periodicity (Fig. 6c, d). One could readily recognize 16 nm repeated features including the different projection densities. Therefore, we next utilized the 16 nm periodicity to box particles of the CPC along the axoneme axis. These sub-volumes centered on the CPC were then aligned to each other, and averaged together with *PEET*, with the procedures described above.

We used the following numbers of particles to generate sub-tomographic averages of various flagellar components: 763 particles from 10 tomograms for DMT, 3001 particles from 52 tomograms for the 16 nm CPC, 558 particles from 52 tomograms for 96 nm CPC, 246 particles from 17 tomograms for each of the PAC4, PAC5, PAC6, and PAC7, 246 particles from 17 tomograms for the PFR proximal zone, 1362 particles from 12 tomograms for the PFR distal zone.

The resolution of each sub-tomographic average was calculated by *calcFSC* in *PEET* based on the 0.143 FSC criterion (Supplementary Fig. S4).

### Sequence alignment and secondary structure prediction of major PFR proteins

For PFR protein sequence alignments, we used PFR1 (NCBI# XP_844025.1) and PFR2 (NCBI# XP_847331.1) from *T. brucei*^59^, PFR-like protein of *Leishmania braziliensis* (NCBI# XP_001565953)^94^, PFR1 (NCBI #AAV53924) of *Angomonas deanei*^95^ from the *Strigomonadinae* family, and PFR 1D (NCBI XP_003872382.1)^96^ of *Leishmania mexicana* from the *Leishmaniinae* subfamily. Sequences were aligned with multiple sequence alignment function in *Clustal Omega* (1.2.4)^97^. For each of the four proteins, secondary structures were predicted using *PSIPRED*^98^. Predicted secondary structures of proteins were combined with protein alignment results to compare the PFR protein structure between different species.

### 3D visualization

*IMOD*^90^ and UCSF *ChimeraX*^99^ were used to visualize reconstructed tomograms and sub-tomographic averages. Segmentation of densities maps was performed by the *volume tracer* and *segger* tools of UCSF *Chimera*^100^. For surface rendering with UCSF *ChimeraX*, maps were first low-pass filtered to either 30 Å or 50 Å. Montage was done in UCSF *ChimeraX* by fitting averaged structures (i.e., 96 nm averaged axoneme, 16 nm averaged CPC, 54 nm averaged PACs, 54 nm averaged PFR proximal zone, etc.) into an unaveraged tomogram after missing-wedge compensation. Fitting of existing model of coiled-coil helix bundle was done with the *molmap* and *fit* functions in UCSF *ChimeraX*^99^. Schematics were drawn by *Adobe Illustrator*. The built-in denoising program in *Warp*^101^ was used to enhance visualization. Tilt series were separated into two sets and reconstructed independently, and the noise will be filtered out to improve the SNR given that the signal is consistent in both maps, but the noise is random.

## Supplementary information

Supplementary Information

Supplementary Movie S1. Density slices through a representative tomogram.

Supplementary Movie S2. 3D montage showing a 1248nm segment of the flagellum axoneme and PFR.

Supplementary Movie S3. Surface view of the averaged PFR distal zone.

Supplementary Movie S4. Surface view of the averaged connections of wire 1 and 5.

Supplementary Movie S5. Surface view of the averaged PFR proximal zone with PAC7 baseplate.

Supplementary Movie S6. Surface view of the averaged CPC.

## Data Availability

The cryoET sub-tomographic averaged maps have been deposited in the EM Data Bank under the accession codes EMD-23619 and EMD-23620 for the PFR distal zone and the central pair complex in *T. brucei*, respectively. A tomogram of the *T. brucei* flagellum in its bloodstream form was deposited in the EM Data Bank under the accession code EMD-23621.
