# Author Correction: Structure of the trypanosome paraflagellar rod and insights into non-planar motility of eukaryotic cells

**DOI:** 10.1038/s41421-021-00317-7

**Published:** 2021-08-06

**Authors:** Jiayan Zhang, Hui Wang, Simon Imhof, Xueting Zhou, Shiqing Liao, Ivo Atanasov, Wong H. Hui, Kent L. Hill, Z. Hong Zhou

**Affiliations:** 1 Department of Microbiology, Immunology and Molecular Genetics, University of California, Los Angeles (UCLA), Los Angeles, CA USA; 2 Molecular Biology Institute, UCLA, Los Angeles, CA USA; 3 California NanoSystems Institute, UCLA, Los Angeles, CA USA; 4 Department of Bioengineering, UCLA, Los Angeles, CA USA

**Keywords:** Cell migration, Cryoelectron tomography

Correction to: *Cell Discovery*

10.1038/s41421-021-00281-2, published online 13 July 2021

In the original publication of this Correspondence^1^, we missed labeling the co-first authors. The first two authors, Jiayan Zhang and Hui Wang, who have contributed to this work equally, should have been designated with a footnote of “These authors contributed equally: Jiayan Zhang, Hui Wang”. This correction does not affect the description of the results or the conclusion of this work.
