# Supplementary material for: Structure of the trypanosome paraflagellar rod and insights into non-planar motility of eukaryotic cells
Source: Cell Discov. 2021 Jul 13;7:51. doi: 10.1038/s41421-021-00281-2 (PMC8277818; doi:10.1038/s41421-021-00281-2)
Supplement: Supplementary file 1 — Supplementary Information [file 41421_2021_281_MOESM1_ESM.pdf]

Supplementary Information

Supplementary figures and figure legends

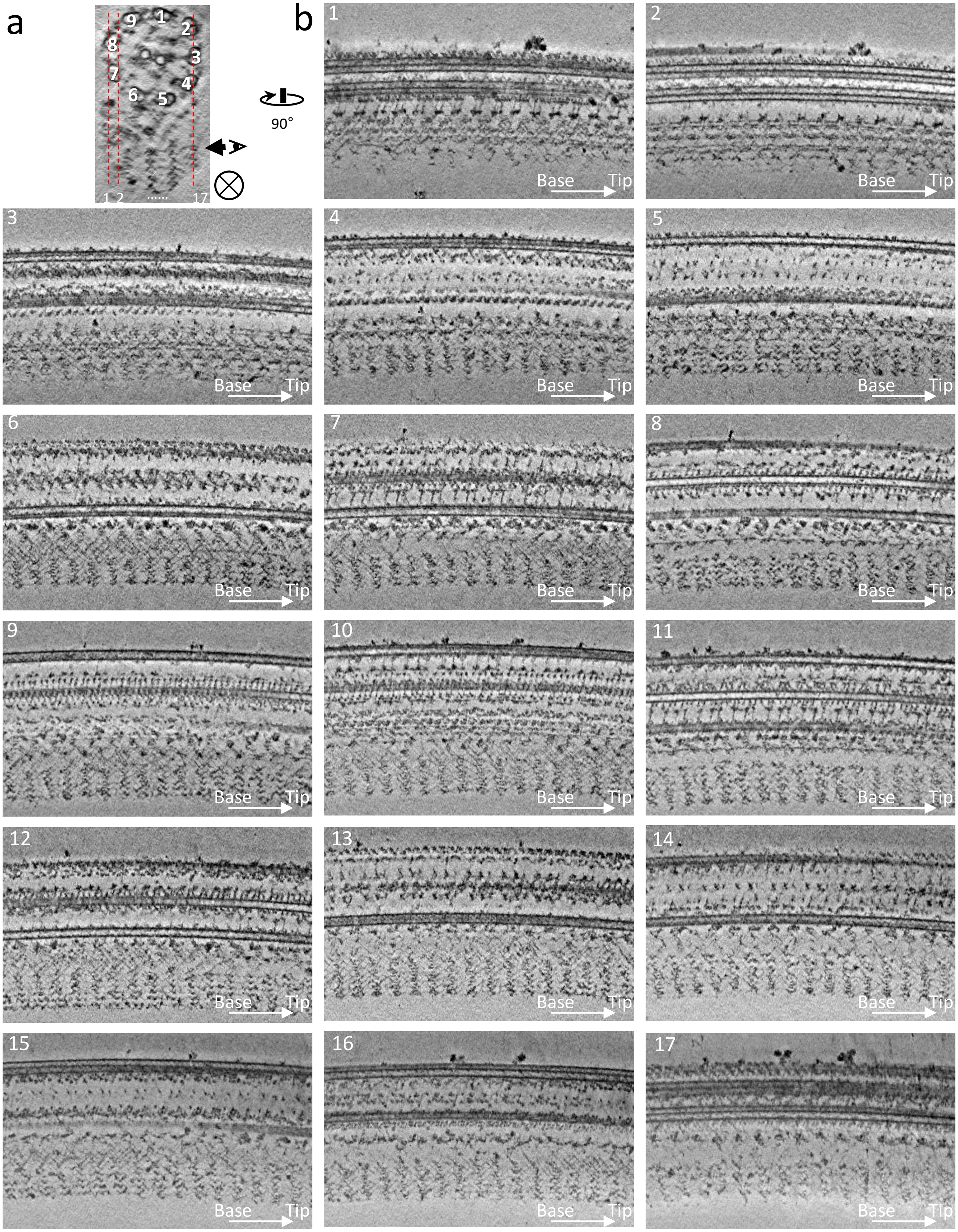

Supplementary Fig. S1. Density slices through a representative tomogram.

(a) Cross section views with DMT numbered.

(b) Seventeen evenly spaced longitudinal sections as indicated by red lines in a.

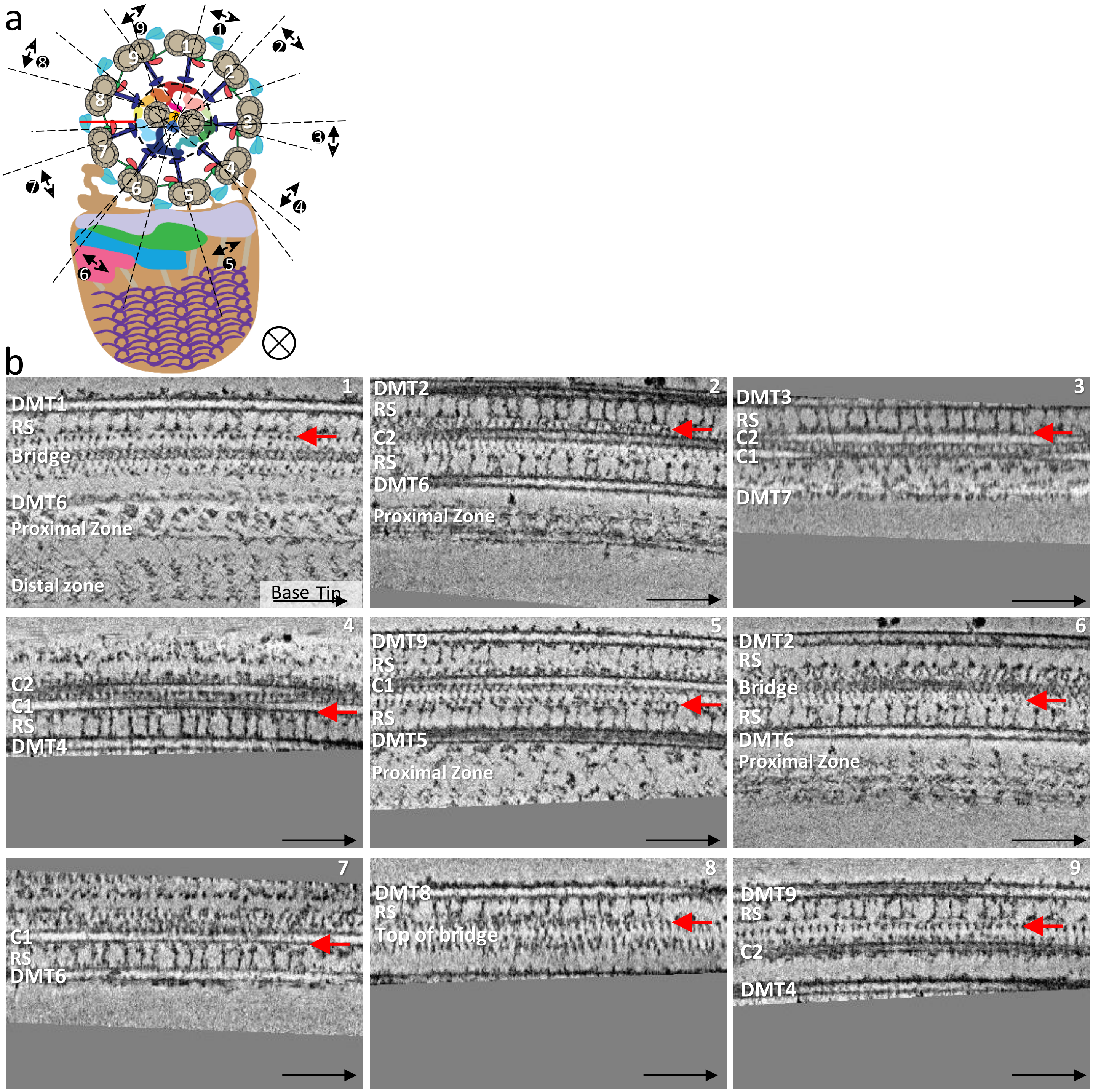

Supplementary Fig. S2. Density slices of a tomogram showing connections between CPC and RS. (a, b) Schematic (a) with nine dashed lines indicating the section planes and viewing directions of longitudinal density slices (b_1-b_9). Arrows indicate connections between the CPC and RS from the indicated DMTs.

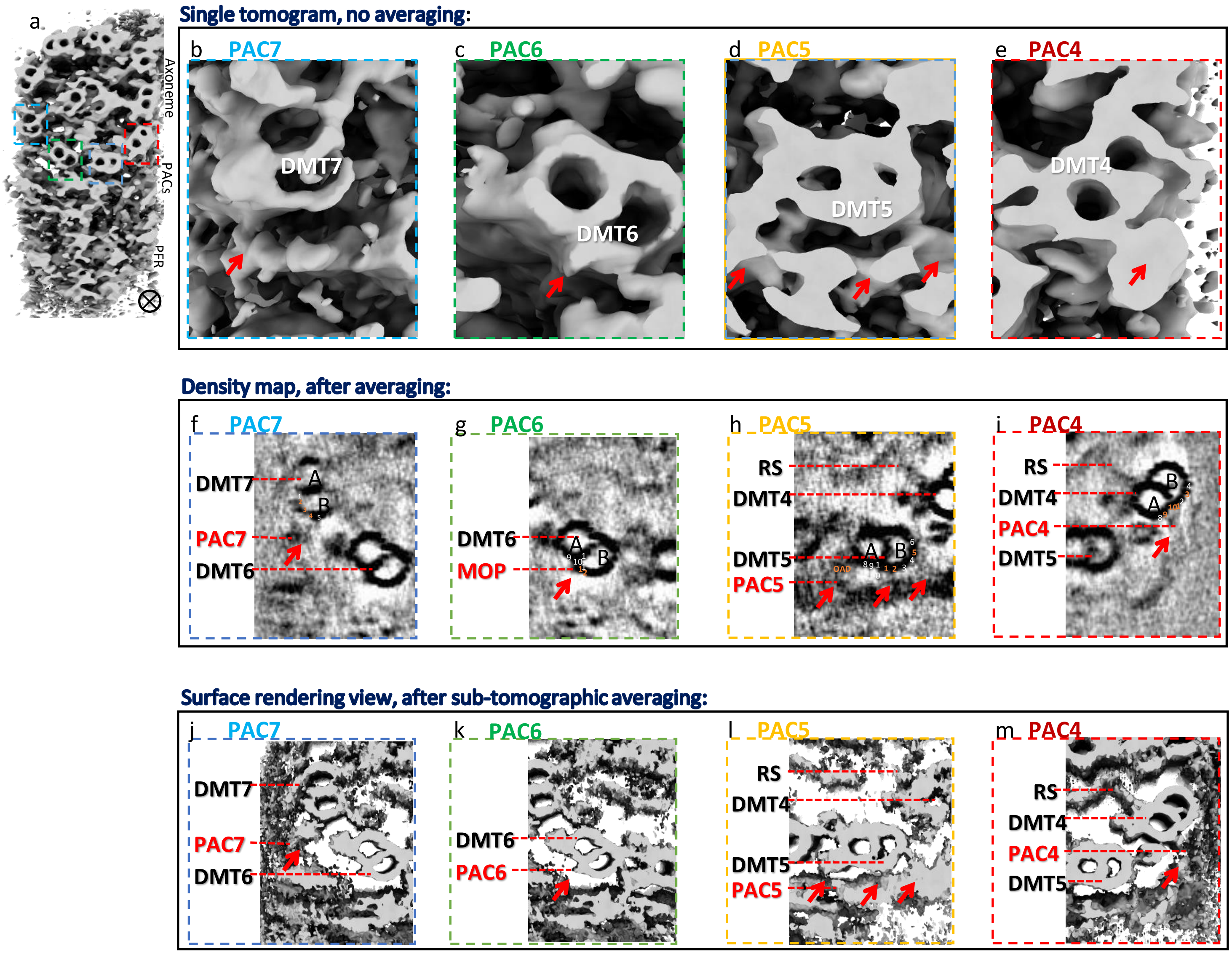

n

PACs	Protofilament / OAD
PAC4	A9, 10; B1, 3
PAC5	OAD | B1, 2 |B5
DMT6-MOP	B1, 2
PAC7	B2-4

Supplementary Fig. S3. PAC contacts with the axoneme.
(a) Surface view of a single tomogram with boxes indicating PAC4-7 regions shown in zoomed-in views in b-e.
(b-e) Surface view of the PAC4-7 regions boxed in panel a from an individual tomogram.
(f-m) Density slices (f-i) and surface views (j-m) of a sub-tomographic average showing regions corresponding to those in b through e, respectively. Protofilaments of the A and B-tubules are numbered according to convention.
(n) Summary of contacts between PACs and the axoneme (see also Fig. 5).

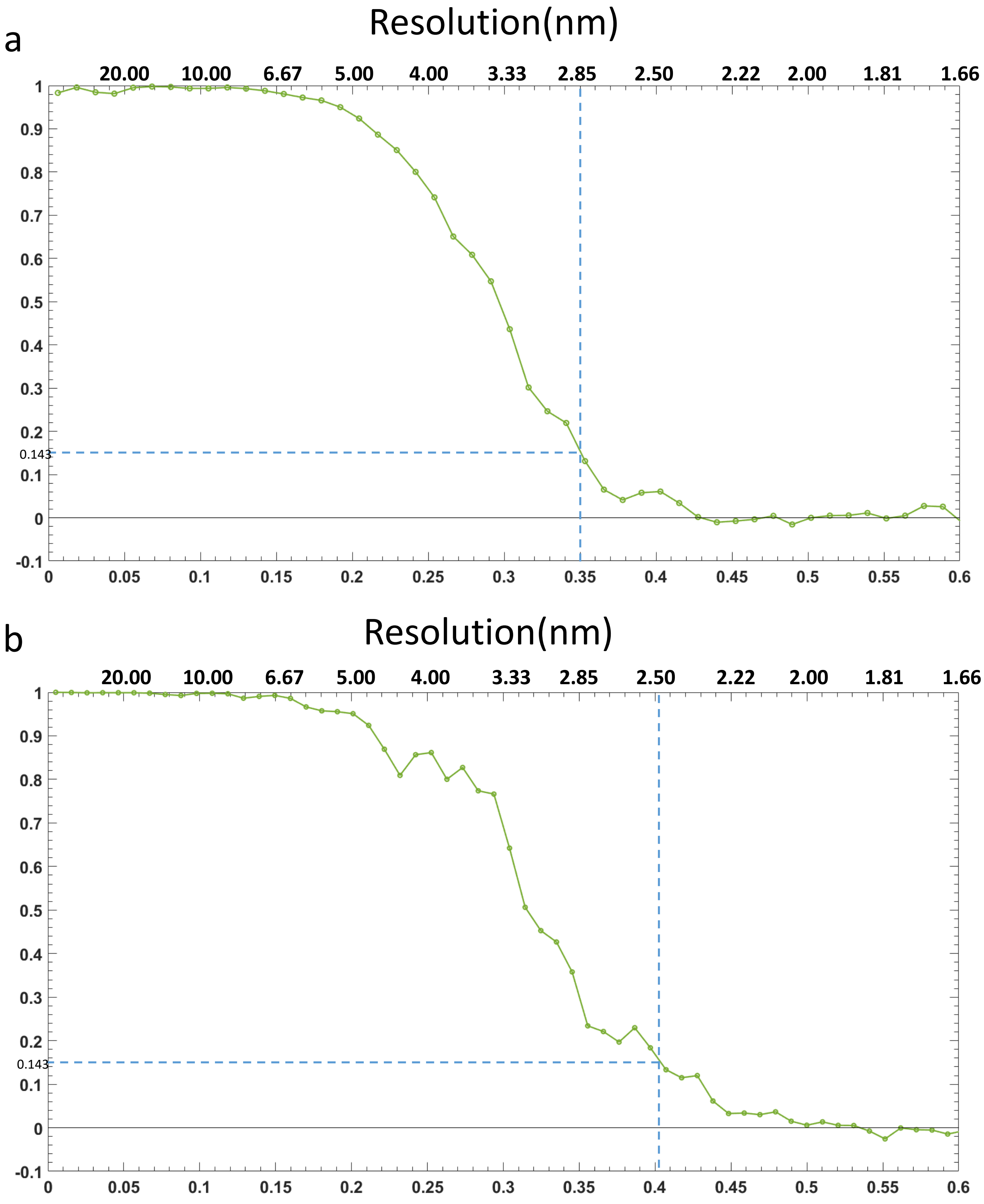

Supplementary Fig. S4. Resolution evaluation of sub-tomographic averages.
(a, b) FSC coefficients as a function of spatial frequency for the final sub-tomographic averages of the distal zone of PFR (a) and the CPC (b). The effected resolution, as indicated on the top of each plot, is based on the FSC at the 0.143 coefficient cutoff.

Supplementary movie titles

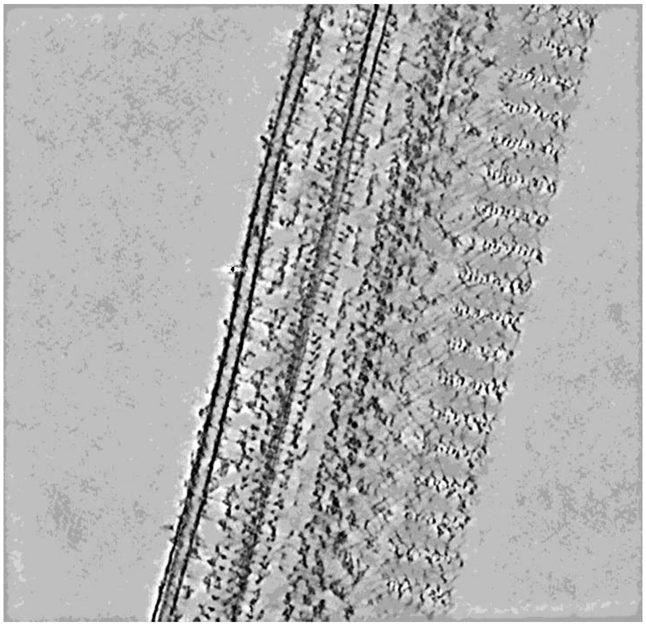

Supplementary Movie S1. Density slices through a representative tomogram.
Slices through a representative tomogram of the T. brucei axoneme and PFR, reconstructed by SIRT and denoised by Warp [Tegunov, D. and P. Cramer (2019). Nat Methods 16(11): 1146-1152]. Related to Fig. 1b. The movie starts with a longitudinal view, similar to that shown in Fig. 1b, but with the flagellum base at the bottom and tip at the top.

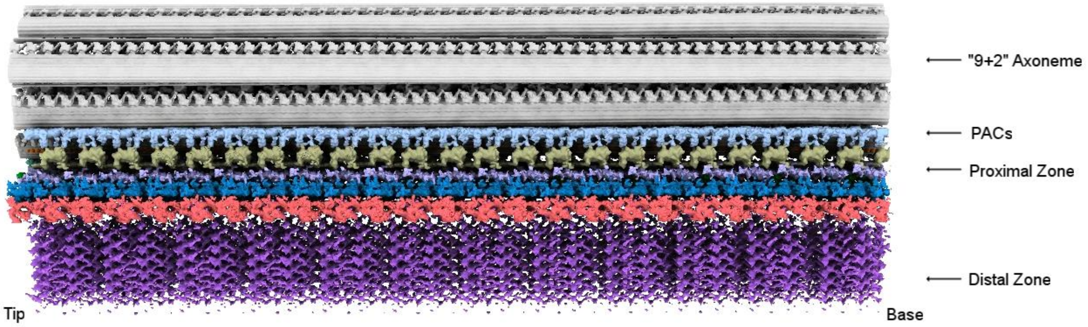

Supplementary Movie S2. 3D montage showing a 1248nm segment of the flagellum axoneme and PFR.
The montage is assembled from individual sub-tomographic averages of axonemal DMTs (from [Imhof, S., et al. (2019). Elife 8]) CPC, PFR proximal and distal zones, and PACs (from this work), positioned relative to each other based on the original tomogram. Tip and base directions are indicated in the movie. Related to Fig. 1d, e. The movie starts view as shown in Fig. 1E, and time point 00:06 corresponds to the view in Fig. 1d.

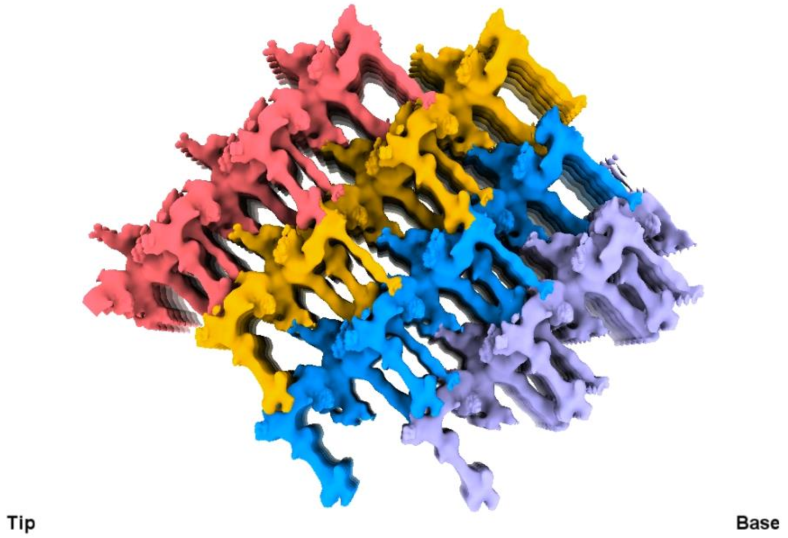

Supplementary Movie S3. Surface view of the averaged PFR distal zone.
Surface view of the averaged PFR distal zone showing four SSN planes (each colored differently) with wires. Related to Fig. 3a-c. The movie starts with view looking at the distal zone from the axoneme side, and corresponds a view tilted ~-45° with respect to Fig. 3a. The views at 00:04s and 00:14s correspond to the view in Fig. 3a. The views at 00:35 and 00:54 correspond to the views in Fig. 3b and 2d, respectively. The view at 00:09s corresponds to the view in Fig. 1d.

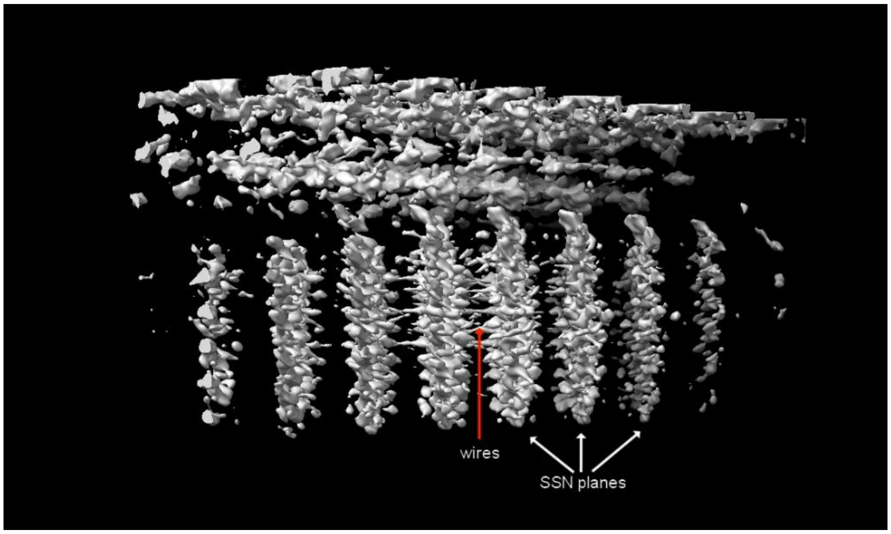

Supplementary Movie S4. Surface view of the averaged connections of wire 1 and 5.
Surface view of the averaged PFR structure showing connections of wire 1 and 5 from the distal zone to densities in the intermediate zone. Related to Fig. 2g and 4b, c. The movie starts with a view corresponding to that shown in Fig. 1e.

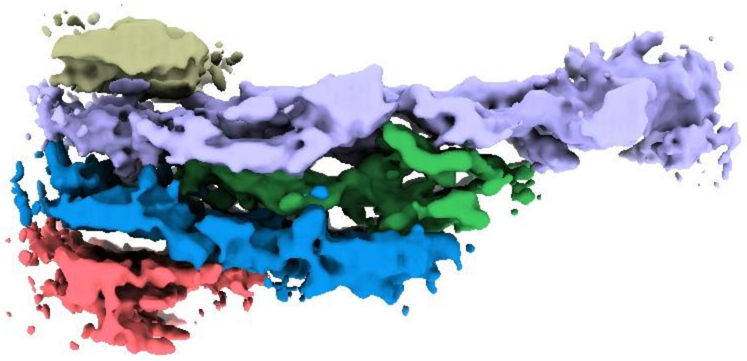

Supplementary Movie S5. Surface view of the averaged PFR proximal zone with PAC7 baseplate.
Surface view of the averaged PFR proximal zone (light purple, green, blue, and pink) with the baseplate of PAC7 (tan). Related to Fig. 4d-i. The movie starts with a view corresponding to that shown in Fig. 1e. The view at 00:23s corresponds to the view shown in Fig. 1d.

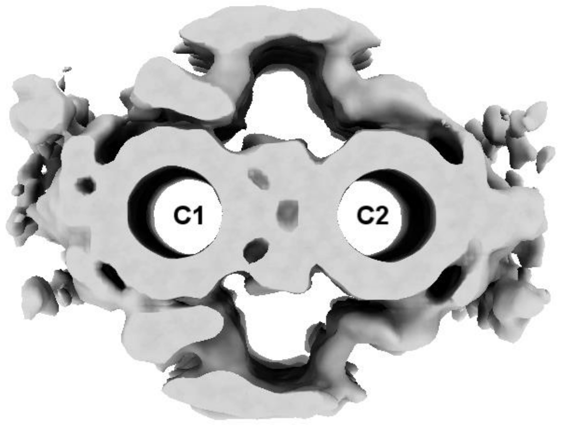

Supplementary Movie S6. Surface view of the averaged CPC.
Surface view of the averaged CPC, showing the C1 and C2 microtubules, projections, and bridge densities. Related to Fig. 6c-e. The movie starts with a view corresponding to that shown in Fig. 1e and 6e. The views at 00:23s and 00:32s corresponds to that shown in Fig. 6c and 6d, respectively. The viewing angle from 00:18s to 00:39s is the same as shown in Fig. 1d.
